# Honey Bee Pollen in Meagre (*Argyrosomus regius*) Juvenile Diets: Effects on Growth, Diet Digestibility, Intestinal Traits, and Biochemical Markers Related to Health and Stress

**DOI:** 10.3390/ani10020231

**Published:** 2020-01-31

**Authors:** Valentina Panettieri, Stavros Chatzifotis, Concetta Maria Messina, Ike Olivotto, Simona Manuguerra, Basilio Randazzo, Andrea Ariano, Fulvia Bovera, Andrea Santulli, Lorella Severino, Giovanni Piccolo

**Affiliations:** 1 Department of Veterinary Medicine and Animal Production, University of Napoli Federico II, Via F. Delpino 1, 80137 Napoli, Italy; valentina.panettieri@unina.it (V.P.); andrea.ariano@unina.it (A.A.); lorella.severino@unina.it (L.S.); giovanni.piccolo@unina.it (G.P.); 2 Institute of Marine Biology, Biotechnology and Aquaculture, Hellenic Centre for Marine Research, Gournes Pediados P.O. Box 2214,71003 Heraklion, Crete, Greece; stavros@hcmr.gr; 3 DiSTeM, Marine Biochemistry and Ecotoxicology Laboratory, University of Palermo, Via G. Barlotta 4, 91100 Trapani, Italy; concetta.messina@unipa.it (C.M.M.); simona.manuguerra@unipa.it (S.M.); andrea.santulli@unipa.it (A.S.); 4 Department of sea science, University Polytechnic of Marche, via Brecce Bianche, 60100 Ancona, Italy; i.olivotto@staff.univpm.it (I.O.); b.randazzo@staff.univpm.it (B.R.); 5 Consorzio Universitario della Provincia di Trapani, Institute of Marine Biology, Via G. Barlotta 4, 91100 Trapani, Italy

**Keywords:** meagre, honey bee pollen, growth trial, digestibility trial, TNF-α, HSP70, intestinal immunohistochemistry, toxic elements, trace elements, total serum protein

## Abstract

**Simple Summary:**

Recently, several studies have focused on the use of nutraceuticals and honey bee products to improve the welfare and sustainability of animal husbandry. Honey bee pollen is rich in bioactive substances, presenting a strong antioxidant activity with possible positive effects on growth performance and non-specific immune responses in reared fish. Despite its favorable characteristics, the addition of honey bee pollen to a meagre (*Argyrosomus regius*) diet in our trial resulted in a reduction of growth performances and diet digestibility, histological alterations of intestinal morphology, and high levels of biomolecular stress markers, probably due to its complex ultrastructure, which is indigestible for monogastric animals. These negative effects could be overcome by using bioactive component extraction methods and thus eliminating the indigestible fractions. Our results confirmed the general assumption that it should always be considered necessary to test nutraceutical additives of natural origin in a given species in order to verify the effective positive action and exclude any negative repercussions on animal health.

**Abstract:**

This research aimed to evaluate the effects of the inclusion of honey bee pollen (HBP) in meagre (*Argyrosoumus regius*) juveniles’ diets on growth performance, diet digestibility, intestinal morphology, and immunohistochemistry. Furthermore, stress-related molecular markers and biochemical blood profile of fish were evaluated, together with mineral trace and toxic element concentration in pollen and diets. Specimens of meagre (360) of 3.34 ± 0.14 g initial body weight, were randomly allocated to twelve 500 L circular tanks (30 fish per tank). Four diets were formulated: a control diet and three experimental diets with 1%, 2.5%, and 4% of HBP inclusion. All the growth parameters and crude protein and ether extract digestibility coefficients were negatively linearly affected by increased HBP inclusion (*p* < 0.0001). Histology of medium intestine showed slight signs of alterations in group HPB1 and HPB2.5 compared to control. Fish from HBP4 group showed severe alterations at the intestinal mucosa level. Immunohistochemical detection of TNF-α in the medium intestine showed the presence of TNF-α+ cells in the lamina propria, which resulted in accordance with the increased level of the TNF-α protein detected by immunoblotting in the liver. This stress situation was confirmed by the increased hepatic level of HSP70 (*p* < 0.05) in fish fed the HBP4 diet and by the linear decrease of total serum protein levels in HBP-containing diets (*p* < 0.0001). These negative effects can be related to the ultrastructure of the bee pollen grain walls, which make the bioactive substances unavailable and can irritate the intestine of a carnivorous fish such as meagre.

## 1. Introduction

In the past 20 years, researchers have focused their attention on several natural molecules to be used as possible antioxidant therapeutic and preventive agents [1].

A growing amount of scientific evidence is demonstrating that supplementing diets with natural compounds that act as protective factors or immunostimulants can improve growth performance, product quality, fish health, and physiological response to stress situations and diseases [2,3,4,5,6].

Bee-derived products have been widely investigated for their positive characteristics, such as their strong antioxidant activity, their positive effects on non-specific immune responses, and their capacity to increase growth performance in various animal species [7,8,9]. Research has been carried out to test the inclusion of these nutraceutical products in aquafeeds because of their antioxidant potential to prevent or treat aquatic animal diseases and to increase their performance [8,10].

Honey bee pollen (HBP) consists of the male generative cells gathered by honeybees from flower stamens and the anthers of flowers, which are collected by foraging bees and carried to the hives, where pollen agglutinates with bee secretions and the addition of nectar [11,12]. The consumption of HBP has increased in the recent years because it has been considered a healthy and therapeutic product because of its nutritional properties, source of proteins, lipids, vitamins, minerals, amino acids, carotenoids, flavonoids, phenolic compounds, antioxidants, carotenes, and xanthophylls [13,14]. Phenolic acid, flavonoids, and tannins act as potent antioxidants and protective agents and their free radical scavenging activity encourages their application in the biomedical field [15,16].

The introduction of HBP in freshwater fish diets has led to improved growth and immune status [17], increase in the number of phagocytic cells, (i.e., neutrophils and monocytes), and reduction of the mortality caused by *Aeromonas hydrophila* in Nile tilapia (*Oreochromis niloticus*) [8].

Despite these favourable characteristics, no research has been carried out to evaluate the effects of the inclusion of HBP in diets for marine fish species. The present research intended to fill this gap, evaluating growth performance, diet digestibility, intestinal morphology and immunohistochemistry, stress-related molecular markers, and biochemical blood profile in meagre (*Argyrosoumus regius*) juveniles fed diets containing increasing levels of commercial HBP. In addition, the research aimed to contribute to the knowledge of the mineral trace element concentration in honey bee pollen and in fish diets.

## 2. Materials and Methods

A chestnut honey bee pollen (HBP) purchased from a local organic farm located in the city of Naples (Napoli, Italy) was chosen for the research. Both growth and digestibility trials were conducted at the Institute of Marine Biology, Biotechnology and Aquaculture (IMBBC) of the Hellenic Centre for Marine Research (Crete, Greece). The experimental protocol was designed according to the guidelines of European Directive (2010/63/EU) on the protection of animals used for scientific purposes. The digestibility trial was approved by the experts of ASSEMBLE Plus project (Contract number: SR020220189047). The growth trial procedures and the blood sampling were approved by ethics experts of the Trans National Access (TNA) selection panel of the AQUAEXCEL 2020 (project number: AE040069).

### 2.1. Growth Trial

#### 2.1.1. Fish and Experimental Conditions

Meagre juveniles were obtained from the IMBBC hatchery. After a 2 week period of acclimation to the experimental conditions, 360 fish of 3.34 ± 0.14 g initial body weight were individually weighed under moderate anaesthesia conditions (anaesthetic: MS222-Tricain Methanesulphonate at 50 mg/L dosage) and randomly allocated to twelve 500 L circular tanks (30 fish per tank) supplied by flow-through borehole aerated seawater (renewal 200% per hour).

The trial was conducted from July to October 2018 under constant temperature (19.0 ± 0.5 °C), salinity (36‰), and dissolved oxygen (DO 8 ± 0.5 ppm) and a dark:light cycle of 12:12 h.

#### 2.1.2. Fish Diets

To determine the effects of the inclusion of HBP, four isonitrogenous (53% crude protein (CP) on as fed basis), isolipidic (15% ether extract (EE) on as fed basis), and isocaloric diets were formulated to meet the meagre nutrient and energy requirements according to Chatzifotis et al. [18]: a control diet with no addition of HBP (HBP0) and three experimental diets in which HBP was included at 1% (HBP1), 2.5% (HBP2.5), and 4% (HBP4) on as fed basis.

The experimental feeds were prepared at the IMBBC laboratory. All dietary ingredients were finely ground and thoroughly mixed; water was then blended into the mixture to obtain an appropriate consistency for pelleting using a 2.5 mm die meat grinder. After pelleting, the diets were dried in a ventilated oven at 40 °C for 24 h and stored in plastic bags until use.

Chemical composition of the experimental diets was determined as follows: dry matter (DM), ash, CP, EE, and crude fiber (CF) (procedure numbers 934.01, 942.05, 954.01, 920.39, and 962.09, respectively) according to AOAC [19]. Before starting the analysis, the pellets were finely ground using a cutting mill. The ingredients and approximate composition of the diets are reported in Table 1 and the approximate composition of the bee pollen is reported in Table 2.

The experimental diets were randomly assigned to triplicate groups of 30 fish.

Each diet was administered three times per day (09.00 am; 12.00 pm, and 4.00 pm) to apparent satiety (until the first pellet was refused), 7 days per week. Any not-ingested pellet was recovered, dried, and weighed. The exact quantity of feed distributed in each tank was recorded daily. The trial lasted 88 days.

#### 2.1.3. Trace Elements

The trace elements contained in the honey bee pollen and in the diets were also determined. Before analysis, the samples (0.5 ± 0.02 g) were placed in a Teflon vessel with 5.0 mL of 65% HNO_3_ and 2.0 mL of 30% H_2_O_2_ (Romil UpA). The vessel was sealed and placed in a microwave digestion system (Milestone, Bergamo, Italy). Microwave-assisted digestion was performed with a mineralization program for 15 min at 200 °C. The vessel was then cooled at 30 °C, the digestion mixture was transferred into a 50.0 mL flask, and the final volume was obtained by adding Milli-Q water [20]. Trace element concentrations were determined by inductively coupled plasma optical emission spectroscopy (ICP-OES) technique using an Optima 2100 DV instrument (PerkinElmer Inc., Wellesley, MA, USA) coupled with a CETAC U5000AT (CETAC Technologies, Omaha, NE, USA). The calibration curve and two blanks were run during each set of analyses, to check the purity of the chemicals. A reference material (CRM DORM-4, National Research Council of Canada (NRC-CNRC), Ottawa (Ontario), Canada) was also included for quality control. All the values of the reference materials were within the certified limits.

The instrumental detection limits are expressed as wet weight (w.w.) and were determined following the protocol described by Perkin Elmer ICP, application study number 57 [21].

#### 2.1.4. Growth Performance

At the end of the trial, fish were starved for 1 day, lightly anesthetized (MS222-Tricain methanesulphonate at 50 mg/L dosage), and group weighed.

The following growth performance indexes and protein efficiency ratios (PER) were calculated according to the following formulas [22]:

- Weight gain (WG%) = 100 × ((FBW, final body weight (g)—IBW, initial body weight (g))/initial live weight (g))
- Daily intake rate (DIR, %/day) = 100 × ((feed intake (g)/mean weight (g))/days)
- Specific growth rate (SGR, %/day) = ((lnFBW − lnIBW)/number of feeding days) × 100
- Feed conversion ratio (FCR) = (total feed supplied (g)/weight gain (g))
- Protein efficiency ratio (PER) = (weight gain (g)/total protein fed (g))

### 2.2. Somatic Traits

At the end of the feeding trial, 45 fish per treatment (15 fish per replicate) were randomly chosen, weighed, and sacrificed by over-anaesthesia (MS222-Tricain methanesulphonate at 250 mg/L dosage) and dissected. Liver and gut were weighed to determine hepatosomatic (HSI) and viscerosomatic (VSI) indexes as described in Piccolo et al. [23]. Liver, muscle, and intestine of four fish for each tank were sampled.

### 2.3. Digestibility Trial

Dry matter, protein, and lipid digestibility of the tested diets was evaluated in a separate trial. One hundred and eighty meagre of 23.53 ± 2.16 g initial weight obtained from the IMBBC hatchery were distributed in 12 circular fiberglass tanks (3 tanks per treatment; 15 fish per tank) of 270 L equipped with a settling column. The water and environmental conditions were the same as described for the growth trial.

Diets were prepared at the IMBBC laboratory following the same procedure described above and using the same inclusion levels of HBP (1%, 2.5%, 4%). The apparent digestibility coefficients were measured using the indirect acid-insoluble ash (AIA) method; for this reason, 1% celite^®^ (Fluka, St. Gallen, Switzerland) was added to the diets used in the growth trial as an inert marker.

Fish were fed the experimental diets ad libitum three times a day for 4 weeks. Feces accumulated in the settling column was collected daily before the morning meal, centrifuged at 7000 rpm for 10 min, and stored at −20 °C until analysis.

Thirty minutes after the last feeding in the afternoon, tanks were cleaned to remove excess of feces, and then the settling column was placed.

Feeds and feces were analyzed according to AOAC [19]. The AIA contents of feeds and feces were determined according to Vogtmann et al. [24].

The apparent digestibility coefficients of dry matter (ADC DM), crude protein (ADC CP), and ether extract (ADC EE) were calculated following Palmegiano et al. [25].

### 2.4. Histology

For the histological analysis, segments of medium intestine were isolated from growth trial fish and immediately fixed in modified buffered Karnovsk fixative for at least 24 h. After fixation, samples were dehydrated in graded alcohol solutions, cleared in xylene, and embedded in solid paraffin. Cross-sections of 5 µm, cut with a LEICA microtome (Leica Microsystems Srl, Buccinasco (MI), Italy), were stained with Mayer’s haematoxylin and eosin Y and examined under a Zeiss Axio Imager.A2 microscope (Carl Zeiss Microscopy GmbH, Jena, Germany); images were acquired by means of a combined colour digital camera Axiocam 503 (Carl Zeiss Microscopy GmbH, Jena, Germany). For the morphometric evaluation of intestinal fold height, and mucous cells abundance, nine fish for each treatment (three for each tank) were considered. Three sections for each fish were observed at intervals of about 200 µm in order to avoid repetitions in morphometric measurements of folds and quantification of mucous cells. Intestinal folds were measured from the apex to the base, excluding the underlying connective layer and values have been expressed by mean and standard deviation (SD).

### 2.5. Immunohistochemistry

For the immunodetection of TNF-α in the medium intestine, nine fish for each treatment (three for each tank) were used and sections (5 µm) were placed on gelatinized slides (0.5% fish gelatin, 0.05% chromopotassium sulfate) in order to prevent section detachment during the reaction. Sections were deparaffinized and rehydrated through serial graded ethanol solutions. Endogenous peroxidase activity was blocked by treating sections with 0.3% hydrogen peroxide for 10 min at room temperature. To prevent aspecific antibody binding, slides were rinsed in 0.01 M phosphate-buffered saline (PBS), pH 7.4, for 15 min and blocked using 5% BSA in Tris for 20 min. After rinsing in PBS for 15 min, sections were incubated overnight at 4 °C with anti-TNF-α rabbit polyclonal primary antibody specific for zebrafish (ANASPEC). After rinsing the slides with PBS, the sections were incubated with the secondary fluorescent antibody (Goat Anti-Rabbit IgG H&L, Alexa Fluor 488 No. ab150077; Abcam, dilution 1:400) at room temperature for 1 h and 30 min. Following incubation, the slides were mounted with Fluoreshield Mounding Medium with DAPI (ab104139) for nucleus staining. The sections were observed under a Zeiss Axio Imager M2 microscope and images were captured with a high-resolution camera Zeiss Axiocam 105 color (Carl Zeiss Microscopy GmbH, Jena, Germany). Negative controls were obtained by incubation without the primary antibody. For the relative quantification of TNF-α+ cells, three sections from three different fish for each treatment were analyzed. Observations were performed counting TNF-α+-labeled cells in three undamaged folds for each section. An arbitrary unit was adopted on the mean of the count: 0–20 = +, 20–50 = ++, 50–>100 = +++.

### 2.6. Extraction of Total Proteins and Detection of Hepatic Biomolecular Markers by Immunoblotting

For total protein extraction, aliquots of lyophilized livers were homogenized on ice (1:3 w/v) with 100 mM HEPES pH 7.4, 10 mM EDTA, 10 mM EGTA, and 4 M NaCl 23.37 g / 100 mL, in the presence of 10 µL protease inhibitor mix (NaF 42 mg/mL, Aprotinine, Na_3_PO_4_ 183.9 mg/mL, Leupeptine 4×). The homogenate was centrifuged at 6000 rpm at 4 °C for 15 min and the supernatant was stored on ice for protein quantification according to Lowry et al. [26]. Biomolecular markers in liver were analyzed by immunoblotting.

Equivalent amounts of proteins (30 μg) diluted with Laemmli buffer (1970) (Sigma-Aldrich, St. Louis, MO, USA) were loaded, after protein denaturation for 5 min at 90 °C, on pre-cast gel for SDS–polyacrylamide electrophoresis (SDS-PAGE) (Bio-Rad, Hercules, CA, USA) and blotted using a Trans Blot Turbo Transfer System (Bio-Rad, Hercules, CA, USA). The correct amount of protein loaded was confirmed by red Ponceau staining. Filters were used for protein detection by primary antibodies (AbI) specifics for TNF-α (Ab polyclonal from rabbit) and HSP70 (Ab monoclonal from rabbit). (Sigma-Aldrich, Dorset, UK; Santa Cruz, CA, USA). The primary antibodies were diluted in buffer at the concentrations suggested by the manufactures for each AbI. In relation to the origin of the AbI, the appropriate secondary antibodies were used (anti-mouse or anti-rabbit secondary); as means of detection, the secondary antibodies were conjugated with horseradish peroxidase (GAR/M-HRP Bio-Rad, Hercules, CA, USA) or alkaline phosphatase. The signals originated by immunoreaction were detected using enhanced chemo-luminescent (ECL) reagents (Clarity Western ECL Blotting Substrate, Bio-Rad, Hercules, CA, USA) and a BCIP/NBT substrate system (Bio-Rad). Images were obtained, photographed, and digitalized with Chemi Doc XRS (Bio-Rad, Hercules, CA, USA), and further analyzed with Image Lab software (Bio-Rad, Hercules, CA, USA). The results were expressed as fold increase of each treatment in relation to the respective control; the images shown are representative of three separate experiments, for which the mean quantification is reported in each figure, together with the significance of the differences (*p* < 0.05).

### 2.7. Assessment of Biochemical Parameters in Blood Samples

Biochemical measurements were performed on blood samples from 2-month-old fish of the species *Argyrosomus regius*, within a weight 20–25 g. The purpose of the test was to evaluate the effect of bee pollen enrichment in accordance with the requirements of the AquaExcel2020 program.

On the day of the sampling, fish were euthanized, and their weight and length was measured. Immediately, blood was collected from the caudal vessel via heparinized syringes. Hematocrit was measured, using capillary tubes and a specialized centrifuge to determine the percentage of red blood cells by volume. Additionally, hemoglobin was determined using a colorimetric assay kit (Spinreact, Girona, Spain).

Following these procedures, blood samples were centrifuged at 2000× *g* for 10 min and the resulting plasma was stored at −20 °C until further analysis. Plasma samples were used to assess the following biochemical parameters: cholesterol, triglycerides, albumin, total proteins, glucose, creatinine, sodium, potassium, calcium, phosphorus, lactate, glutamic oxaloacetic transaminase (GOT), and glutamic pyruvic transaminase (GTP).

More specifically, plasma cholesterol (CO/PAP, Biosis, Greece), triglycerides (GPO/PAP, Biosis, Athens, Greece), albumin (BCG, Biosis, Greece), total serum proteins (Biuret, Biosis, Greece), glucose (GOD/PAP, Biosis, Greece), calcium (Arsenazo III, Biosis, Greece), phosphorus (UV, Biosis, Greece), lactate (LO-POD, Spinreact, Girona, Spain), and creatinine (Cayman Chemical Company, Ann Arbor, MI, USA) concentrations were assessed using commercial enzymatic colorimetric kits. Moreover, GOT-AST (IFCC/LIQUID, Biosis, Greece), GPT-ALT (IFCC/LIQUID, Biosis, Greece) concentrations were estimated using enzymatic kinetic assays. Finally, a flame-photometer was used to determine plasma sodium and potassium concentrations.

### 2.8. Statistical Analysis

The data were tested for normal distribution and equal variances before analysis (SAS, 2000, SAS Institute Inc., Cary, NC, USA). All the data were analyzed by one-way ANOVA, using the GLM procedure of SAS (2000), according to the model

Yij = m +Di + eij,(1)

For growth and digestibility trials, the experimental unit was the tank and each value was obtained as the average of the 30 fish in the tank. For somatic indexes, the experimental unit was the individual fish (15 fish for each tank). Finally, for the blood parameters, the unit was derived from the average of four fish per tank.

In addition, the mean comparison was performed using orthogonal contrast analysis. The examined components were linear and quadratic (SAS, 2000, SAS Institute Inc., Cary, NC, USA). *P* < 0.05 was considered the threshold for statistical significance.

## 3. Results

### 3.1. Trace Elements

Table 3 shows the content of toxic and essential trace elements in experimental feeds and honey bee pollen.

### 3.2. Growth Performance

In Table 4, the growth parameters measured during the trial are reported.

All the growth parameters were significantly influenced by the diet. Daily feed intake increased as bee pollen inclusion in the diet increased (*p* < 0.01), while final weight (*p* < 0.0001), FCR (*p* < 0.001), SGR (*p* < 0.01), PER (*p* < 0.001), and WG %ABW (*p* < 0.01) were negatively linearly affected by bee pollen inclusion in the diet. The growth parameters reached the worst values in the HBP4 diet.

### 3.3. Somatic Indexes, Slaughter Traits

In Table 5, the viscerosomatic indexes and the slaughter traits are shown.

No significant differences in somatic indexes emerged among the fish fed the experimental diets, except for the gut length, which showed a linear increase with increasing honey bee pollen inclusion in the diet.

### 3.4. Digestibility Trial

The estimated ADC of DM, CP, and EE of the four diets are reported in Table 6.

ADC DM linearly decreased as the HBP content in the diets increased. Furthermore, linear and quadratic components indicated a gradual reduction of crude protein and ether extract digestibility coefficients going from the control to HBP4 diet. Nevertheless, the ADCs of HBP1 diet resulted higher than those shown by HBP2.5 and HBP4 groups, as indicated by the quadratic component of the variance (*p* < 0.001).

### 3.5. Histology

Histology of the medium intestine showed slight signs of alterations in group HPB1 and HPB2.5 when compared with the HBP0 group. No significant differences were shown by the morphometric evaluation of intestinal folds among experimental groups (Table 7).

**Figure 1:**
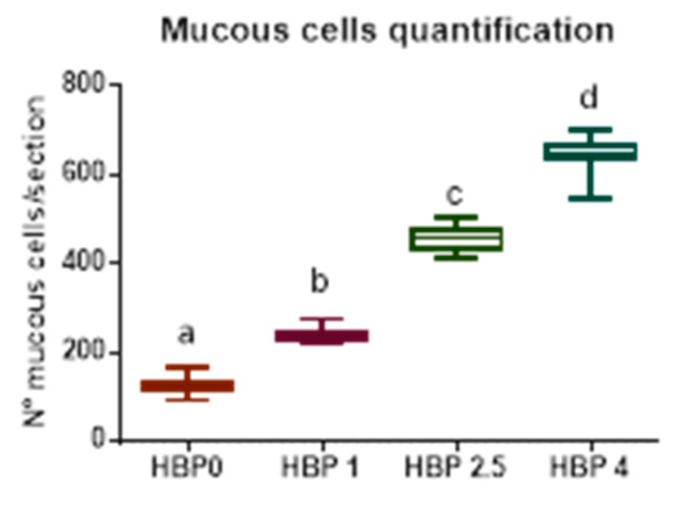
Abundance of mucous cell in medium intestine. The number of mucous cells is reported as mean of the observations performed on three transversal intestinal sections from individual fish (n = 9) from the different experimental groups. Different letters indicate significant differences among groups (*p* < 0.05).

A significant linear increase in mucous cells in response to pollen inclusion was observed (Figure 1). 

**Figure 2:**
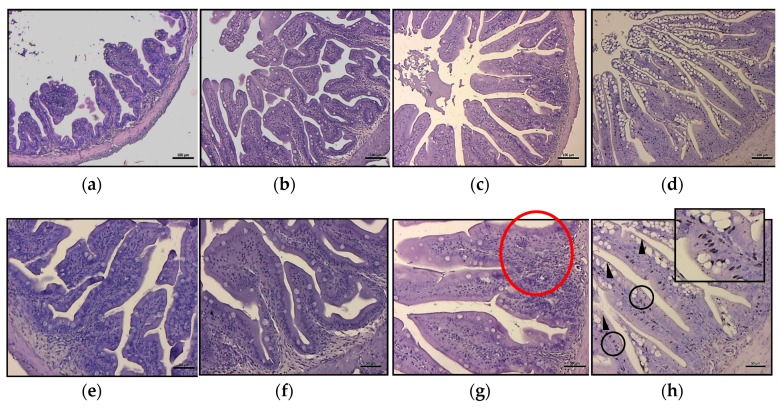
Intestinal histology of meagre fed experimental diets after 88 days of trial. Histology of *A. regius* medium intestine from the different experimental groups: (**a**,**e**) normal histology from HBP0 group; (**b**,**f**) HBP1 group did not show signs of pathological alteration; (**c**,**g**) in the HBP2.5 group, it was possible to detect inflammatory influx in some portions of the intestinal submucosa (red circle); (**d**,**h**) the HBP4 group showed a severe degree of mucipar hyperplasia of the mucosa (arrowhead) with an abundant presence of melano macrophages at the base of the enterocytes (black circle and box). Scale bar: a, b, c, d = 200 µm; e, f, g, h = 50 µm.

The most frequently encountered alterations consisted of leucocyte infiltration at the level of the lamina propria and thickening of the submucosa (Figure 2g). Intestine samples from HBP4 group showed severe alteration at the level of the mucosa, even preserving epithelial integrity. In this group, a substantial increase in the number of mucous cells was observed (Figure 1 and Figure 2). Moreover, melano macrophage-like, melanin-containing cells were often observed at the base of the epithelial layer and infiltrating lamina propria and submucosa (Figure 2h).

### 3.6. Immunohistochemistry

**Figure 3:**
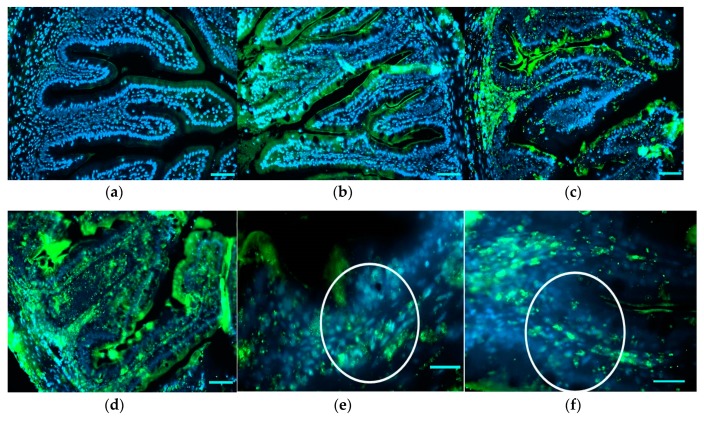
Immunohistochemical detection of TNF-α in medium intestine of the different experimental groups (**a–d**). High magnification images showing TNF-α+ cells (white circle) in the submucosa and in the lamina propria of intestine from HBP2.5 (**e**) and HBP4 (**f**) groups. Positive reactions are shown in green. Nuclei were stained with DAPI (blue). Scale bar: a,b,c,d = 50 µm; e,f = 20 µm.

Immunohistochemical detection of TNF-α in medium intestine showed the presence of TNF-α+ cells in the lamina propria and submucosa of bee-pollen-treated fish. While a weak reaction was recorded in the epithelial layer of intestinal folds in all treated groups, a moderate influx of TNF-α+ cells was recorded in particular in groups HBP2.5 and HBP4 (Figure 3). In particular, round macrophage/neutrophil-like TNF-α+ cells were observed in the submucosa and lamina propria of HBP1, HBP2.5, and HBP4 groups. The relative abundance evaluation of TNF-α+ cells infiltrating submucosa and lamina propria showed low values in HBP1, while HBP2.5 and HBP4 showed high values with no significant differences between the two groups (Table 8) at the base of the epithelial layer and infiltrating lamina propria and submucosa.

### 3.7. Hepatic Biomolecular Markers

**Figure 4:**
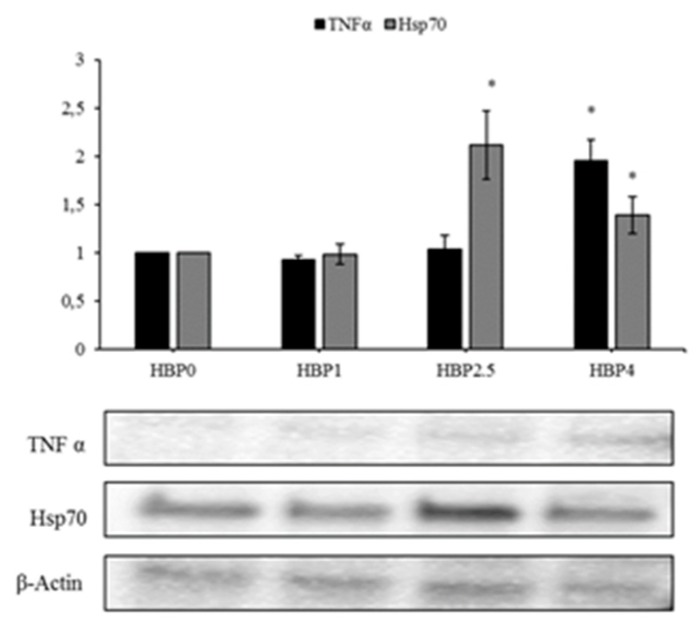
Immunoblot of TNFα, HSP70, evaluated on samples of the liver of *A. regius* fed on three different diets containing increasing levels of commercial HBP (HBP1, HHBP2.5, HBP4) and in standard condition (HBP0). β-Actin was used as the internal control. The images are representative of at least three separate experiments. The relative protein quantification is represented in the graphic (* *p* < 0.05 vs. HBP0).

The evaluation of the molecular markers related to immunostimulation and stress resistance showed that the levels of the protein TNF-α presented a significant increase only in fish fed the diet with the highest level of HBP inclusion (HBP4) (*p* < 0.05), while HSP70 resulted increased both in HBP2.5 and HBP4 groups compared to the control group (Figure 4).

### 3.8. Assessment of Blood Samples

The results obtained from the blood analysis are shown in Table 9.

This table highlights the differences among the groups. Total serum protein, globulin, and albumin to globulin ratio linearly and quadratically decreased as the HBP content in the diets increased.

## 4. Discussion

The trial results showed that honey bee pollen induced negative effects on meagre growth performance and diet digestibility. These data were not in line with the current, although limited bibliography, in which bee pollen had notable beneficial effects in other fish species, as described by El-Asely for Nile tilapia and Choobkar for rainbow trout [8,10], even if it must be remarked that these latter authors used a bee pollen alcoholic extract.

The bad results obtained with the in vivo results can be explained by looking at the other performed investigations. The intestinal histology evidenced pathological alteration of the mucosa morphology, showing a severe mucipar hyperplasia and an abundant presence of melano macrophage-like cells at the base of the enterocytes. These alterations became more evident as the inclusion of bee pollen in the diets increased, where, besides the inflammatory influx, the migration and infiltration of melanin-containing cells indicated a further, extreme index of an immune response. The presence of TNF-α cells, resembling in shape neutrophil cells or macrophages, in fish fed different HBP inclusion corroborated the histological results. Tumor necrosis factor-alpha is an important pro-inflammatory cytokine playing an important role in cell survival through the activation, proliferation, and differentiation of macrophages. It is released by several immune cells during infection and tissue damage and its inhibition increases susceptibility to disease and reduces capacity to resolve infection [27,28,29,30]. TNF-α isoforms have been identified in various fish species [27,28,31], showing high levels of conservative regions with mammals and a constitutive expression in healthy fish tissues [28]. Inflammation is a highly energy-demanding process and it has been widely shown that intestinal inflammation correlated to anti-nutritional factors in fish diet is able to affect growth in fish [32,33].

Regarding the variation of the liver biomolecular markers related to general stress response and immunostimulation, in this case the increase of TNF-α could also be ascribed to a significant production of mediators of inflammation, acting as a strategy of protection [34]. The positive modulation of TNF-α has been observed in specimens of *Cyprinus carpio* treated, during vaccination, with *Aloe vera*, and also on immunity response in poultry, mice, and humans [35]. In that case, the effects were attributed to some poorly defined compounds of the plant [35].

Members of the HSP70 family are widely used as biomarkers of environmental stress in ecological and toxicological studies in fish [36,37,38]. The functions of the different HSP70 family members depend on their cellular localization, acting both as chaperons and immunomodulators [38], with the general aim of maintaining protein homeostasis [39] in normal and stressful conditions. HSP70 can be induced in response to thermal stress, hypoxia, oxidative stress, ultraviolet radiation, nutrient deprivation, osmotic pressure, heavy metals, chemical agents, microbial infections, and inflammation [37,38,39,40,41].

The levels of HSP70 protein increased in fish fed with the highest doses of bee pollen inclusion (HBP2.5 and HBP4) (Figure 4), indicating a stress condition that could be related to metabolic or oxidative stress. In fact, this protein is normally present at a low level in organisms with a good balance of antioxidants and over-expressed in a situation of oxidative stress [42,43,44]. Our previous study on the inclusion of dehydrated lemon peel in the diet for seabream showed a reduction of the HSP70 levels in fish fed on increased levels of bioactive compounds, due to the antioxidant composition of this resources, which is able to guarantee protection against oxidative stress [2]. Accordingly, Di Giancamillo et al. [45] described a decrease of HSP70 in pigs fed a diet supplemented with a natural verbascoside extract, compared with pig fed a high-fat diet. These authors suggested a role of HSP70 in modulating hepatic oxidative stress [45].

The main evidence that emerged from the blood analyses was the linear reduction of the total serum protein levels, concomitant with the increase of HBP inclusion in diets. TSP is a non-destructive parameter that is robust, easy to measure everywhere, and cheap, representing a suitable way of monitoring the overall welfare of fish by its regular increase [46]. It has been reported that this parameter tends to decrease in fish in response to various stress conditions such as hyperoxia, hypercapnia, stocking density, transfer to another tank, or nodavirus injection [46]. Many explanations have been suggested to explain TSP decrease, such as changes in blood volume and plasma hydration, alterations in permeability of environmental barriers, tissue destruction, malabsorption, and tissue injury by parasites or other pathogens [47]. These results confirm what emerged in the intestinal histology and immunohistochemistry and in hepatic-stress-related biomolecular markers.

Given that the fish of the different groups were subjected to the same environmental conditions, and received isoproteic and isoenergetic diets that varied only according to the level of pollen inclusion, our results can be explained through a negative action of the pollen in terms of intestinal inflammation.

Concerning the mineral content, the macro elements and the trace element contents in our HBP were in line with the values reported by Taha [48]. Compared to the maximum levels (MLs) of heavy metals set by the EU Commission (Directive (EC) No 2002/32 [49]), Cd, Pb, and as levels in honey bee pollen were lower than the maximum values established for feedstuff and feed materials, indicating that the risk of exposure to heavy metals deriving from consumption of the honey bee pollen used in this trial was relatively low and in compliance with EU regulations. However, in all the diets used in the trial, the levels of potentially toxic elements resulted lower than the MLs stated by EU regulation establishing the following MLs of heavy metals content in mg/kg (ppm) relative to a feed with a moisture content of 12%: the Cd MLs in the feed materials and complete feed are 2.0 and 0.5 mg/kg, respectively; the Pb MLs in the feed materials and complete feed are 10.0 and 5.0 mg/kg, respectively; the As MLs in the feed materials and complete feed are both 2 mg/kg. These elements, therefore, may not have been important in determining the observed deterioration of the growth performance and health status of the intestine in such fish species.

To deeply comprehend the reason for such negative results, it is necessary to dwell on the microscopic structure of bee pollen. Honey bee pollen is composed of a multitude of microscopic particles (6–200 μm in diameter) with variable shape, usually spherical or oval. The pollen cell walls consist of a series of stratified concentric layers [50]. As Roulstone described, the outermost layer of the pollen wall is the pollenkitt [51], a semi-solid coating comprised primarily of neutral lipids, hydrocarbons, terpenoids, and carotenoid pigments [52]. Inside the pollenkitt is the exine, an often intricately ridged matrix of the complex carbohydrate sporopollenin. Sporopollenin is a compound that provides chemical resistance to bee pollen and preserves the compounds within it [53]. The chemical analysis of sporopollenin has been limited due to the difficulty of obtaining large quantities of exine without affecting or modifying the structure of this molecule. Atkin et al. [53] recently found that this molecule has an empirical formula C_90_H_144_O_27_. Nowadays, it is suggested that the sporopollenin consists of a series of biopolymers, homologous to the compounds cutin, suberin, and lignin.

The exine greatly resists decay and digestion, but is commonly perforated by one-to-several pores or slits (germination pores) that lead to the inner wall layer, known as the intine. The intine, composed primarily of cellulose and pectin, is also very resistant, and forms the final barrier to the nutrient-rich cytoplasm. From reports based on literature, it is known that the exine is rich in antioxidant compounds, while the intine has the rest of the nutritional and bioactive compounds [54]. Thus, any animal consuming pollen consumes pollenkitt nutrients through external probing of pollen grains or ingestion of pollen grains, but must penetrate or dismantle two resistant pollen wall layers in order to access cytoplasmic nutrients [51]. As the pollen has such complex ultrastructure, it is not certain that the beneficial substances contained in it are available for monogastric animals. Previous studies have shown that the exine and intine are indigestible by the human digestive tract [55]. Furthermore, administering pollen suspended in water to mice by means of gastric intubation resulted in grains being found in the feces [56]. The artificial digestion at various pH values and after various incubation times appeared unaffected after acid treatment (corresponding to gastric digestion), with the exception of the digestion of substances located outside the walls. Partial digestion of grains occurred only during alkaline treatment in the presence of pancreatic enzymes [57]; grains may be partially emptied if the enzymes are able to penetrate the exine pores, and it depends also on the thickness of the intin layer.

Taking into account the difficulties of monogastric animals in digesting bee pollen grains and the similarity of pollen walls with indigestible fibrous substances such as suberin and lignin, it can be assumed that bee pollen had a pro-inflammatory action on the intestinal mucosa of meagre, a fish with carnivorous feeding habits [19,58]. In contrast, the bee pollen structure may not represent a problem to herbivorous fish species such as the Nile tilapia, with an enzymatic profile more suitable for the digestion of fibrous substances and in which positive effects of honey bee pollen addition to the diet have been reported [8].

## 5. Conclusions

In conclusion, the results of the present trial showed that the addition of honey bee pollen to juvenile meagre diets not only did not have the positive effects described in the literature for other fish species, but it even had negative effects on both growth performance and diet digestibility. Moreover, the inflammatory state of the intestine progressively worsened as the level of HBP inclusion increased. These effects could be ascribed to the ultrastructure of the bee pollen grain walls (exine and intine), which made the bioactive substances unavailable for the fish, while the intestinal inflammation could have been due to the bee pollen grains’ chemical composition rich in lignin- and suberin-like substances, which can irritate the intestine of monogastric animals such as carnivorous fish. These negative HBP effects could be overcome by using an extraction method able to concentrate the bioactive substances and eliminate the indigestible fractions. As a general conclusion, it is worth highlighting that it should always be considered necessary to test nutraceutical additives of natural origin in each species in order to verify the effective positive action and exclude any negative repercussions on animal health.

## Figures and Tables

**Table 1:** Ingredients and chemical composition of experimental diets for meagre.

Ingredients (g/kg)	HBP0	HBP1	HBP2.5	HBP4
Fish meal	440	438	435	430
Soybean meal	240	238	235	230
Corn gluten meal	100	98	96	95
Wheat flour	100	98	93	90
Fish oil	100	98	96	95
Honey bee pollen	0	10	25	40
Mineral mix	10	10	10	10
Vitamin mix	10	10	10	10
Chemical Composition				
Dry matter (%)	90.0	92.7	90.6	93.0
Ash (%)	9.4	9.8	9.0	9.4
Crude protein (%)	53.0	53.6	52.5	52.5
Ether extract (%)	15.0	14.9	15.0	14.8
Crude fiber (%)	7.7	7.7	7.7	7.5
Na (%)	0.086	0.075	0.058	0.075
Mg (%)	0.130	0.117	0.131	0.125
K (ppb)	381.65	351.92	381.93	377.90
Ca (ppb)	870.39	696.49	456.83	562.36

**Table 2:** Chemical composition of honey bee pollen.

	DM (%)	Ash (%)	CP (%)	EE (%)	CF (%)	Na (%)	Mg (%)	K (%)	Ca (%)
HBP	85.02	3.79	23.18	7.82	19.23	0.067	0.062	0.019	0.061

Abbreviations: HBP: honey bee pollen; DM: dry matter; CP: crude protein; EE: ether extract; CF: crude fiber.

**Table 3:** Toxic and essential trace elements in experimental feeds and honey bee pollen.

Trace Elements (ppb)	HBP0	HBP1	HBP2.5	HBP4	Honey Bee Pollen
Fe	499.27	490.53	511.96	512.95	134.63
Cu	40.17	38.13	44.73	38.86	6.16
Zn	186.06	178.50	193.95	191.28	29.77
Co	0.15	0.15	0.15	0.15	0.02
Al	62.98	46.01	56.82	66.21	58.13
Se	0.55	0.53	0.54	0.57	1.85
Mn	259.04	262.06	257.79	268.78	31,73
Pb	0.22	0.23	0.23	0.24	0.16
Cd	0.12	0.10	0.11	0.11	0.03
Cr	0.81	0.79	0.83	0.82	0.30
Ni	1.92	1.81	1.90	1.88	0.34
As	2.45	2.23	2.41	2.35	0.59

**Table 4:** Growth performance of meagre fed experimental diets.

	HBP0	HBP1	HBP2.5	HBP4	*p*-Values			
					RMSE	Linear ^1^	Quadratic ^1^	Cubic ^1^
Initial BW (g)	3.35	3.34	3.34	3.34				
Final BW (g)	26.78	23.29	22.69	21.36	0.66	<0.0001	<0.0001	0.0659
FCR	0.96	1.04	1.18	1.23	0.06	0.0007	0.0003	0.3186
SGR	2.37	2.21	2.18	2.11	0.08	0.0044	0.0062	0.4158
Intake‰ ABW/d	16.48	17.23	19.08	19.43	0.98	0.0061	0.0025	0.336
WG%	705.3	606.9	579.7	539.7	52.9	0.0050	0.0059	0.5564
PER	1.96	1.80	1.61	1.55	0.10	0.0008	0.0004	0.5619

Abbreviations: Initial BW: initial body weight; Final BW: final body weight; FCR: feed conversion ratio; SGR: specific growth rate; Intake‰ ABW/d: intake per kg adjusted body weight; WG%: weight gain percentage per initial body weight; PER: protein efficiency ratio. ^1^ Contrast analysis.

**Table 5:** Somatic indexes of meagre fed experimental diets.

	HBP0	HBP1	HBP2.5	HBP4	*p*-Values			
					RMSE	Linear ^1^	Quadratic ^1^	Cubic ^1^
Final Weight	37.4	33.9	36.8	33.9	4.41	0.0584	0.316	0.037
VSI (%)	4.37	4.27	4.20	4.11	0.70	0.3299	0.333	0.9185
HIS (%)	1.62	1.69	1.67	1.72	0.36	0.4245	0.517	0.6901
K	1.17	1.05	1.06	1.06	0.13	0.0397	0.078	0.3916
Gut length %BW	37.50	45.64	40.09	44.05	7.46	0.0413	0.282	0.0206

Final weight: final body weight; HIS: hepatosomatic index; VSI: viscerosomatic index; K: condiction factor; ^1^ Contrast analysis.

**Table 6:** Digestibility trial results.

	HBP0	HBP1	HBP2.5	HBP4	*p*-Values			
					RMSE	Linear ^1^	Quadratic ^1^	Cubic ^1^
ADC DM	64.24	74.28	36.03	45.63	0.82	<0.0001	<0.0001	0.0001
ADC CP	90.26	91.36	78.71	78.56	0.76	<0.0001	<0.0001	0,0003
ADC EE	93.45	92.15	85.725	85.91	0.91	<0.0001	<0.0001	<0.0001

ADC DM: dry matter apparent digestibility coefficient; ADC CP: crude protein apparent digestibility coefficient; ADCEE: ether extract apparent digestibility coefficient. ^1^ Contrast analysis.

**Table 7:** Morphometric evaluation of mucosal folds.

	MF Height (µm)
HBP0	462.3 ± 18.7
HBP1	487.8 ± 23.5
HBP2.5	453.1 ± 24.8
HBP4	476.6 ± 46.4

MF height: mucosal fold height. Data are expressed as mean and SD.

**Table 8:** Relative density of TNF-α+ cells in medium intestine.

CTRL	HBP1	HBP2.5	HBP4
+	+	+++	+++

**Table 9:** Biochemical assessment of blood samples.

	HBP0	HBP1	HBP2.5	HBP4	*p*-Value			
					RMSE	Linear ^1^	Quadratic ^1^	Cubic ^1^
HCT (%)	28.67	26.00	27.00	26.67	3.19	0.46	0.62	0.56
HGB (g/dL)	3.83	3.80	3.76	3.56	0.40	0.43	0.45	0.88
Glucose (mmol/L)	4.71	5.21	4.92	5.96	1.20	0.24	0.34	0.51
TSP (g/dL)	10.20	8.15	6.53	3.73	0.98	<0.0001	<0.0001	0.58
Albumin (mg/dL)	1.61	1.91	1.38	1.64	0.60	0.94	0.60	0.33
Globulin (mg/dL)	8.56	5.72	5.14	2.07	1.27	0.0004	0.0007	0.2331
A/G	0.19	0.43	0.36	0.83	0.24	0.01	0.03	0.27
Creatinine (µmol/L)	80.00	132.50	94.67	104.33	16.51	0.16	0.76	0.03
GOT (UI/L)	360.00	345.00	355.67	243.33	67.7	0.11	0.16	0.48
GPT (U/L)	335.50	298.50	273.33	386.67	49.45	0.30	0.48	0.41
Triglyceride (mmol/L)	13.83	16.43	15.10	17.10	2.45	0.14	0.28	0.28
Cholesterol (mg/dL)	6.63	7.63	9.55	6.90	0.91	0.73	0.19	0.07
P (mg/dL)	7.57	9.50	9.77	11.90	4.63	0.29	0.32	0.78
Ca (mg/dL)	27.07	30.87	22.53	33.47	7.31	0.32	0.75	0.14
Na (mmol/L)	210.33	179.67	206.67	187.00	24.35	0.27	0.67	0.14
K (mmol/L)	2.70	2.17	2.07	2.53	0.73	0.79	0.75	0.95
Lactate (mmol/L)	3.17	4.15	2.75	3.3	0.73	0.83	0.45	0.11

HCT: hematocrit; HGB: hemoglobin; TSP: total serum protein; A/G: albumin to globulin ratio; GOT: glutamic oxaloacetic transaminase; GTP: glutamic pyruvic transaminase. statistically different. ^1^ Contrast analysis.
